# Assessment of Compound-Specific Fatty Acid δ^13^C and δ^2^H Values to Track Fish Mobility in a Small Sub-alpine Catchment

**DOI:** 10.1021/acs.est.2c02089

**Published:** 2022-07-21

**Authors:** Matthias Pilecky, Libor Závorka, David X. Soto, Fen Guo, Leonard I. Wassenaar, Martin J. Kainz

**Affiliations:** † WasserCluster Lunz—Biologische Station, Inter-University Center for Aquatic Ecosystem Research, Dr. Carl-Kupelwieser Promenade 5, 3293 Lunz am See, Austria; ‡ Donau-Universität Krems, Department for Biomedical Research, Dr. Karl-Dorrek-Straße 30, 3500 Krems, Austria; § International Atomic Energy Agency, Isotope Hydrology Section, Vienna International Centre, A-1400 Vienna, Austria; ∥ Guangdong Provincial Key Laboratory of Water Quality Improvement and Ecological Restoration for Watersheds, Institute of Environmental and Ecological Engineering, Guangdong University of Technology, Guangzhou 511458, China; ⊥ University of Saskatchewan, Department of Geological Science, 114 Science Place, Saskatoon SK S7N 5E2, Canada

**Keywords:** carbon stable isotopes, compound-specific isotope analysis, hydrogen stable isotopes, fatty acids, fish migration, site specificity

## Abstract

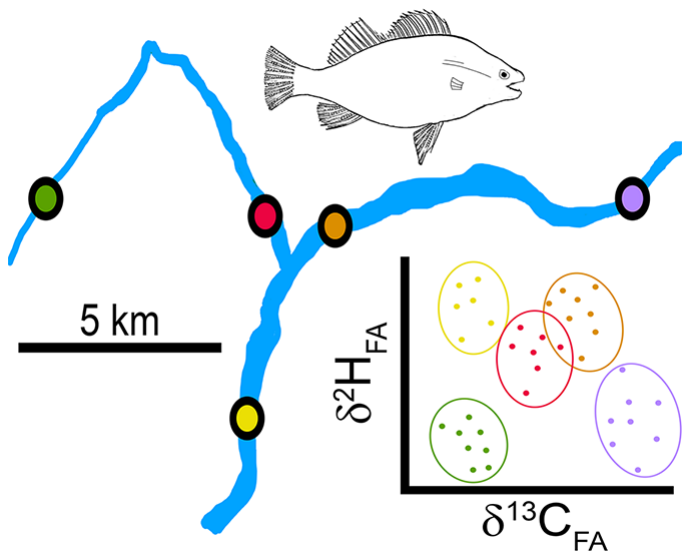

Methods for identifying origin, movement, and foraging areas of animals are essential for understanding ecosystem connectivity, nutrient flows, and other ecological processes. Telemetric methods can provide detailed spatial coverage but are limited to a minimum body size of specimen for tagging. In recent years, stable isotopes have been increasingly used to track animal migration by linking landscape isotope patterns into movement (isoscapes). However, compared to telemetric methods, the spatial resolution of bulk stable isotopes is low. Here, we examined a novel approach by evaluating the use of compound-specific hydrogen and carbon stable isotopes of fatty acids (δ^2^H_FA_ and δ^13^C_FA_) from fish liver, muscle, brain, and eye tissues for identifying site specificity in a 254 km^2^ sub-alpine river catchment. We analyzed 208 fish (European bullhead, rainbow trout, and brown trout) collected in 2016 and 2018 at 15 different sites. δ^13^C_FA_ values of these fish tissues correlated more among each other than those of δ^2^H_FA_ values. Both δ^2^H_FA_ and δ^13^C_FA_ values showed tissue-dependent isotopic fractionation, while fish taxa had only small effects. The highest site specificity was for δ^13^C_DHA_ values, while the δ^2^H isotopic difference between linoleic acid and alpha-linolenic acid resulted in the highest site specificity. Using linear discrimination analysis of FA isotope values, over 90% of fish could be assigned to their location of origin; however, the accuracy dropped to about 56% when isotope data from 2016 were used to predict the sites for samples collected in 2018, suggesting temporal shifts in site specificity of δ^2^H_FA_ and δ^13^C_FA_. However, the predictive power of δ^2^H_FA_ and δ^13^C_FA_ over this time interval was still higher than site specificity of bulk tissue isotopes for a single time point. In summary, compound-specific isotope analysis of fatty acids may become a highly effective tool for assessing fine and large-scale movement and foraging areas of animals.

## Abstract 2

Compound-specific stable isotopes of fatty acids in fishes predict site-specific feeding grounds and track metabolic processes of essential dietary nutrients.

## Introduction

Knowledge of fish migration and movement is central to understanding of processes at individual and population levels over various temporal (e.g., seasons) and spatial (e.g., spawning and feeding grounds) scales.^1^ Migration ultimately plays an essential role in connectivity, adaptation, and gene flow among populations.^2,3^ Movement of fish within or among stream ecosystems at various scales is important for ecosystem functioning including nutrient transfer, food web structure,^4^ and bioturbation or substrate erosion.^5,6^ Hydropower and flow regulation (e.g., dams and weirs) often create physical barriers to migrating fish, disconnecting important spawning, nursery, and foraging sites and affecting the continuum of stream habitats.^7^ In addition, pollution, eutrophication, and habitat loss pose a global threat to stream ecosystems.^8,9^ Thus, new approaches for quantifying the large- and small-scale movement patterns of potamodromous fishes and identifying their breeding and feeding grounds are required for deriving environmentally informed conservation and management strategies.^10,11^

Several methods are currently used to track migration and movement of fishes. Mark-recapture methods require tagging and recapture of the same individuals or detection in close range (i.e., in case of transponder tags), and are hence biased to the location of marking effort, and so do not yield detailed information about finer-scale habitat use by individuals.^12^ Telemetric studies using satellite, acoustic, or radio telemetry can provide fine spatial and vector detail but are limited by the body size and number of individuals.^13^ More recently, intrinsic spatial markers, such as stable isotopes, DNA barcoding, and fatty acid (FA) profiles have been successfully applied.^14^ Methods using stable isotopes of animal tissues (e.g., ^2^H, ^13^C, ^15^N, ^18^O, ^34^S, ^86/87^Sr) are fundamentally based on exploiting larger-scale spatial isotope patterns caused by temperature, altitude gradients, ecozone, or biogeochemical processes.^15^ Isotopic techniques allow for unbiased sampling because no recapture is required and hence provide important information about where individuals acquired their dietary energy to build their tissues. Bulk tissue isotope analyses (e.g., muscle, fins, and scales) are widely used to investigate larger-scale animal migration in terrestrial, marine, and freshwater ecosystems,^1,16−19^ however, bulk isotope methods rely on the presence of distinctive isotopic gradients amongst migratory habitats and usually over larger distances, that is, 100–1000’s of km (but see ref (19)). Using ^86/87^Sr, it was possible to identify the spawning sites of salmon with over 90% accuracy in a large river catchment covering several 100 km.^20^

Finer-scale spatial resolution for isotope provenance assignment might be achieved by using compound-specific isotope analysis (CSIA), as shown by using δ^13^C and δ^15^N in amino acids.^21^ Depending on the metabolic requirements of individuals, a combination of dietary source acquisition and metabolic processes may lead to spatially explicit δ values of essential and non-essential components that ultimately depend on the isotopic composition and food web structure of the local environment in space and time.^22^ Fatty acids, whose δ^2^H and δ^13^C isotopic composition can potentially provide more detailed smaller spatial resolution than bulk tissue stable isotope methods, have never been tested or exploited for animal migration and provenance studies.^23^

Several δ^13^C (bulk tissue) gradients have been identified so far, for example, in correlation with moisture content for terrestrial plants,^24^ as well as a positive increase of 1–2‰ km^–1^ altitude in terrestrial grazers,^25^ hummingbirds,^26^ and terrestrial leaves.^27,28^ Temperature and *p*CO_2_ are suspected to be the main driver of increasing δ^13^C-DIC values by altitude, however, soil respiration rate, instream metabolism, and anaerobic respiration cumulatively contribute to significant spatial variations in δ^13^C-DIC as well^29^ and subsequently influence bulk and compound-specific δ^13^C values of primary producers and consumers.^30^

The aim of this study was to measure patterns of FA carbon and hydrogen stable isotope values in fish tissues at small spatial scales (1–5 km) to assess their potential for studying fish migration and movement ecology and to evaluate the temporal isotope value stability. We evaluated sampling location (site specificity) as variation factor of δ^2^H and/or δ^13^C of FA in tissues (liver, brain, eyes, and muscle tissues) of three fish species (European bullhead, *Cottus gobio*; brown trout, *Salmo trutta*; and rainbow trout, *Oncorhynchus mykiss*) along reaches of a sub-alpine stream catchment in 2016 and 2018. In addition, bulk FA stable isotope values from same fish species were also examined. Bullheads generally have low migration range, rarely move beyond several hundred meters, and hence should exhibit high isotopic site specificity.^31^ In contrast, salmonids like brown trout can migrate distances of 100 km or more,^32^ but in small mountainous catchments are also often stationary,^33^ and thus could exhibit high or low isotopic site specificity.

## Methods

### Study Area

**Figure 1:**
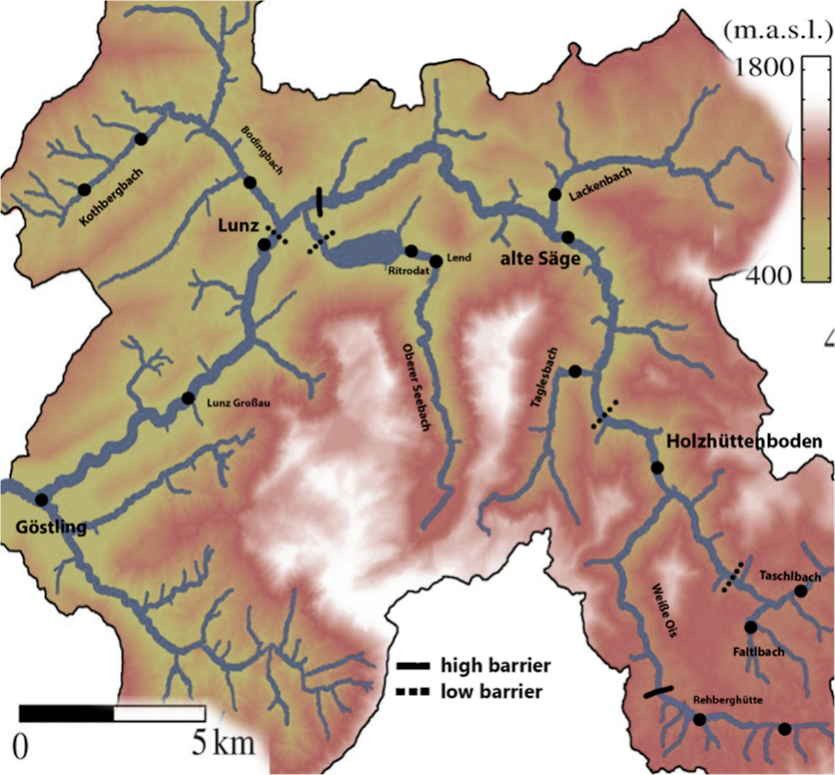
Map of the river Ybbs catchment (47° 84′ 50″ N, 15° 81′ 20″ E) with fish sampling sites. Thick lines indicate impassable (high) migration barriers. Dashed lines indicate low impact migration barriers such as fish ladders or weirs that might be seasonally passable.

The study area was the sub-alpine River Ybbs catchment, Austria (47° 84′ 50″ N, 15° 81′ 20″ E, Figure 1), a small watershed with a drainage area of ca. 254 km^2^. The River Ybbs tributaries and mainstem are first to fifth order streams (Strahler). The maximum distance between fish sampling sites was 35 km between “Weiße Ois” and “Göstling” (Figure 1), which are the highest and lowest altitude sites in the catchment at 1070 and 525 m a.s.l., respectively. Fish migration or connectivity is disrupted in several places in the watershed by natural and manmade obstacles. Detailed descriptions of the study catchment with its sampling sites, including water chemistry parameters are reported in detail elsewhere.^34^

For δ^13^C and δ^2^H analyses of FA in tissues, fish (*n* = 159 in 2016; *n* = 49 in 2018) were sampled and analyzed from 15 sites of 10 streams during base-flow conditions (fall season, Figure 1). Samples collected at the same sites in two different years allowed us to conduct a first-order temporal evaluation of site-specific repeatability of FA stable isotope data. Three fish species (*O. mykiss*, *S. trutta*, and *C. gobio*) were collected by electrofishing and euthanized in accordance with the Austrian Federal Act on the Protection of Animals (http://www.ris.bka.gv.at). All fish were dissected for samples of dorsal muscle and liver, eyes, and brain for FA analysis. Detailed analysis of fish tissue lipid composition for our samples was reported previously.^35^

### Gas Chromatography and δ^13^C and δ^2^H Analyses of FA

Lipids of the selected tissues were extracted as described by Heissenberger et al. (2010). Briefly, freeze-dried tissue samples were homogenized and mixed with chloroform/methanol (2:1 vol/vol) following sonication, and vortexed and centrifuged three times to remove non-lipid materials. The solvent-extracted lipids were evaporated to a final volume of 1.5 mL under N_2_. For fatty acid methyl ester (FAME) formation, lipid samples were incubated with a sulfuric acid/methanol mixture (1:100 vol/vol) for 16 h at 50 °C, following the addition of KHCO_3_ and hexane. Samples were then shaken, vortexed, and centrifuged, and the upper organic layers were collected, pooled, and concentrated under N_2_.^36^

All δ^13^C and δ^2^H analyses of FA were conducted following the analytical methodology described previously.^23^ Briefly, a Thermo Trace 1310 GC (ThermoFisher Scientific, Waltham, MA) was connected via a ConFlo IV (ThermoFisher Scientific) to an isotope ratio mass spectrometer (IRMS, DELTA V Advantage, ThermoFisher Scientific). FAMEs were separated using either a VF-WAXms 60 m column, 0.25 mm ID, film thickness 0.25 μm; or a VF-WAXms 30 m column, 0.32 mm ID, film thickness 1 μm (both Agilent, Santa Clara, CA) and then for δ^13^C analysis oxidized to CO_2_ in a combustion reactor, filled with Ni, Pt, and Cu wires, at a temperature of 1000 °C, or for δ^2^H analysis reduced to H_2_ by passing through a high thermal conversion reactor kept at 1420 °C. Due to better isotope-ratio mass spectrometry detection limits for CO_2_ gas compared to H_2_, the total number of FA peaks identified for δ^2^H was less than for δ^13^C, which however, did not affect our analysis.

Samples were reference scale-normalized using three-point calibration with certified FAME stable isotope (Me-C20:0) standards (USGS70: δ^13^C = −30.53‰, δ^2^H = −183.9‰, USGS71: δ^13^C = −10.5‰, δ^2^H = −4.9‰, and USGS72: δ^13^C = −1.54‰, δ^2^H = +348.3‰), which were also used to check and correct for instrumental drift and linearity. The δ^13^C and δ^2^H values of individual FAME were determined by automated peak integration by defining 0.5 mV/s as the start and end point of a FAME peak and by using dynamic background removal calculation. All peaks were validated and corrected manually if necessary. FA δ^13^C and δ^2^H values (δ*I*_FA_) were corrected for the methyl group addition during methylation according to the formula^23^
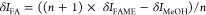
where δ*I*_FAME_ are the δ^2^H or δ^13^C values of the measured FAME and δ*I*_MeOH_ the δ^2^H or δ^13^C values of the methanol used during methylation, and *n* equals the total number of H-/C-atoms of the FAME molecule. Values for δ^13^C are referenced to Vienna PeeDee Belemnite
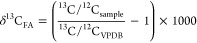


Values for δ^2^H are standardized against Vienna Standard Mean Ocean Water
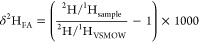


Integrated “bulk” FA values for both δ^2^H (δ^2^H_FAbulk_) and δ^13^C (δ^13^C_FAbulk_) were calculated by summing the isotopic value by the mass fraction of all identified peaks of a sample and divided by the sum of their mass fractions. These composite values were used as proxies for “bulk lipid” stable isotope values for comparison with individual FA.

### Data Analysis

Data and graphical analyses were performed in R (version 4.0.2) using the packages *rstatix*, *ggplot2*, *ggpubr*, *lme4*, *car*, *CCA*, *ccp*, *candisc*, *rptR*, *mass*, and *Morpho*. A Shapiro–Wilks test was used to affirm normal distribution of the data. Significance of the linear models was evaluated using ANOVA using Type II and III sums of squares for models without and with significant interaction terms, respectively, thereby controlling for tissue type, sampling location, and taxa where necessary. For post-hoc univariate analysis of compound-specific effects, *p*-values were adjusted for simultaneous inference by term using the Holm method.^37^ Estimated marginal means for post-hoc group comparisons were calculated using the emmeans package and its pairs() function while applying Šidák adjustments. Canonical correlation analysis (CCA) was performed (CCA package) using the δ^13^C and δ^2^H values of FA as covariates. Significance of dimensions was tested using the *ccp* package and Wilks’ Lambda *F*-approximation. Canonical coefficients (ccoef) were calculated using the *candisc* package.

Site specificity of FA and δ^13^C and δ^2^H data was analyzed by performing canonical variate analysis using the cva() function of the *Morpho* package and linear discriminant analysis (LDA) provided by the *mass* package. Overall, site classification accuracy is given before and after (in parenthesis) cross-validation by running 999 bootstrapping analyses. Site-specific repeatability, describing the relative partitioning of variance into within-group and between-group sources of variance,^38^ was calculated for each isotope and FA compound individually, while controlling for other group variables (e.g., taxa and organ) using the *rptR* package with 1000 bootstrapping analyses.

Unless otherwise mentioned, individual δ values are reported as the mean ± standard deviation, while site-specific isotopic values are provided as estimated marginal means [lower and upper 95% confidence interval].^39^

## Results

### Variation in FA Isotopic Data by Compound, Site, Taxa, and Tissue Type

**Figure 2:**
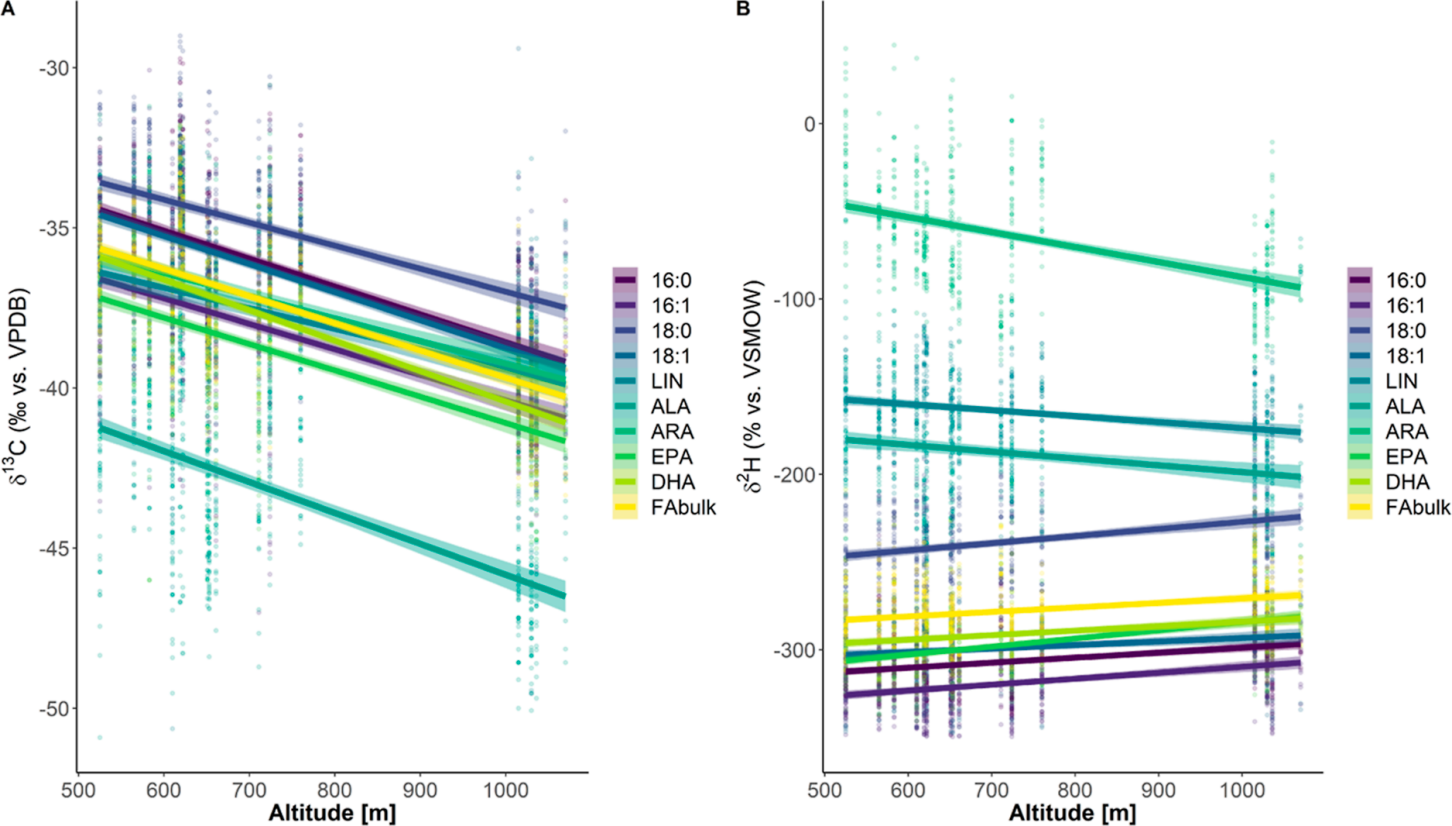
δ^2^H and δ^13^C values of FA in fish samples by sampling site altitude. A strong correlation was observed for δ^13^C, which showed all fatty acids getting isotopically lighter with altitude. No significant correlation with altitude was observed for δ^2^H values of fatty acids, with some fatty acids showing weak positive or negative correlations.

For the evaluation of sampling site-specific FA stable isotope values (δ^13^C_FA_ and δ^2^H_FA_), we used fish samples (*n* = 159; including 64 bullheads, 36 rainbow trout, and 59 brown trout) of the larger and comprehensive 2016 data set to ensure robust results. For this data set, 10,138 GC-IRMS FAME peaks were used for δ^13^C and 9,183 for δ^2^H analysis (Figure 2). In this pooled data set, FA compounds explained most of the variation for both isotopes (δ^13^C: *F*_1,79,111_ = 285.7, *p* < 0.001; δ^2^H: *F*_1,77,982_ = 2199.8, *p* < 0.001). The FA δ^13^C values differed significantly across sampling sites (*F*_1,59,111_ = 216.8, *p* < 0.001) and tissue types (*F*_39,111_ = 59.4, *p* < 0.001) and to a lesser degree across fish taxa (*F*_29,111_ = 11.0, *p* = 0.00002). The δ^2^H_FA_ values were predominantly influenced by fish taxa (*F*_27,982_ = 52.8, *p* < 0.001) and tissue type (*F*_37,982_ = 41.6, *p* < 0.001) and to a lesser extent by the sampling site (*F*_1,57,982_ = 5.8, *p* < 0.001). In a post-hoc analysis (ANOVA, Holm method), all FA compounds showed significant (unless otherwise mentioned; *p*.adj < 0.001) sampling site-specific differences for both δ^13^C_FA_ and δ^2^H_FA_. The tissue types also explained some of the variation in compound-specific δ^13^C_FA_ and δ^2^H_FA_ values, except for the *n* – 6 PUFA ARA and LIN, and additionally for EPA and ETA for δ^13^C_FA_. Significant taxa-specific influence of δ^13^C values was only observed for 16:1 and ALA (*p*.adj = 0.019), while δ^2^H had taxa-specific differences in 14:0, 16:0, 16:1 (*p*.adj = 0.025), 18:0, LIN (*p*.adj = 0.020), ALA (*p*.adj = 0.037), ARA (*p*.adj = 0.023), 20:3*n* – 3, ETA, DPA, and DHA. Hydrogen isotope differences among taxa were found mainly between bullheads and salmonids, while the only significant difference between rainbow and brown trout was observed in δ^2^H_ARA_ (−40.4‰ [−46.4; −34.4] vs. −69.1‰ [−73.3; −65.0], Šidák, *p*.adj < 0.001). Large hydrogen isotopic differences between bullheads and salmonids were observed for δ^2^H and δ^13^C values of 20:3*n* – 3, ETA, and DPA. No significant correlation or patterns between isotope values and physiological parameters such as size, weight, or Fulton’s *K* were identified.

As individual FA compounds were the most important factors explaining variation in δ^2^H and δ^13^C, a lipid “bulk” isotopic value was calculated for each fish sample to assess the contribution of each compound to bulk ^2^H and ^13^C variations (Table S1). Correlations among δ^13^C_FA_ versus δ^13^C_FAbulk_ were significantly higher compared to the corresponding δ^2^H values (mean Pearson correlation vs FA_Bulk_ δ^13^C: 0.732 ± 0.142; δ^2^H: 0.391 ± 0.378; Table 2).

**Table 1:** Group-Specific Assignment of Fish Based on Canonical Variate Analysis of CSIA Data without and with Considering Bootstrapping Cross-Validation

			CVA-cluster		+bootstrap CV	
	tissue		site-specific	≤1 mismatch	site-specific	≤1 mismatch
*C. gobio*	muscle	(*n* = 59)	55 (93%)	57/59 (97%)	47 (80%)	52/59 (88%)
	brain	(*n* = 56)	54 (96%)		51 (91%)	
	liver	(*n* = 47)	43 (91%)		37 (79%)	
	eye	(*n* = 52)	52 (100%)		42 (81%)	
*O. mykiss*	muscle	(*n* = 30)	28 (93%)	29/30 (97%)	25 (83%)	24/30 (80%)
	brain	(*n* = 29)	27 (93%)		19 (66%)	
	liver	(*n* = 17)	17 (100%)		14 (82%)	
	eye	(*n* = 26)	21 (81%)		18 (69%)	
*S. trutta*	muscle	(*n* = 59)	57 (97%)	55/59 (93%)	51 (86%)	48/59 (81%)
	brain	(*n* = 58)	56 (97%)		48 (83%)	
	liver	(*n* = 39)	38 (97%)		27 (69%)	
	eye	(*n* = 54)	51 (94%)		43 (80%)	
			CSIA-accuracy		FA_bulk_-accuracy	
			CVA	bootstrap-CV	CVA	bootstrap-CV
overall	muscle	(*n* = 148)	93.20%	73.60%	38.50%	34.50%
	brain	(*n* = 143)	92.70%	71.50%	43.10%	37.10%
	liver	(*n* = 103)	94.40%	69.40%	38.90%	33.30%
	eye	(*n* = 132)	91.40%	73.60%	40%	37.90%

**Table 2:** Pearson Correlation of CSIA with Fatty Acid Bulk Values and Site-Specific Repeatability

	correlation with bulk		site-specific repeatability δ^13^C				site-specific repeatability δ^2^H			
	*r*^13^C	*r*^2^H	brain	eye	liver	muscle	brain	eye	liver	muscle
14:0	0.734	0.313	0.33 ± 0.11	0.61 ± 0.12	0.16 ± 0.09	0.60 ± 0.12	0.59 ± 0.12	0.45 ± 0.12	0.37 ± 0.13	0.33 ± 0.11
16:0	0.925	0.778	0.71 ± 0.10	0.61 ± 0.12	0.62 ± 0.12	0.81 ± 0.08	0.50 ± 0.12	0.42 ± 0.12	0.38 ± 0.13	0.44 ± 0.12
16:1	0.700	0.696	0.63 ± 0.11	0.45 ± 0.12	0.38 ± 0.13	0.56 ± 0.12	0.41 ± 0.12	0.33 ± 0.12	0.31 ± 0.13	0.15 ± 0.08
18:0	0.851	0.833	0.60 ± 0.12	0.59 ± 0.12	0.49 ± 0.14	0.59 ± 0.12	0.39 ± 0.12	0.35 ± 0.11	0.52 ± 0.13	0.12 ± 0.07
18:1	0.908	0.826	0.74 ± 0.10	0.71 ± 0.10	0.45 ± 0.14	0.70 ± 0.10	0.30 ± 0.11	0.32 ± 0.12	0.23 ± 0.11	0.20 ± 0.09
LIN	0.610	0.217	0.37 ± 0.12	0.55 ± 0.13	0.38 ± 0.13	0.43 ± 0.12	0.19 ± 0.09	0.32 ± 0.11	0.27 ± 0.12	0.59 ± 0.12
ALA	0.645	–0.122	0.56 ± 0.12	0.65 ± 0.11	0.59 ± 0.13	0.76 ± 0.09	0.43 ± 0.12	0.58 ± 0.12	0.47 ± 0.13	0.67 ± 0.11
SDA	0.443	0.217	0.62 ± 0.12	0.37 ± 0.12	0.26 ± 0.12	0.25 ± 0.10	0.18 ± 0.12	0.18 ± 0.09	0.13 ± 0.10	0.10 ± 0.07
ARA	0.635	–0.449	0.52 ± 0.12	0.63 ± 0.12	0.40 ± 0.13	0.59 ± 0.12	0.38 ± 0.12	0.40 ± 0.12	0.46 ± 0.14	0.42 ± 0.12
ETA	0.630	0.392	0.58 ± 0.12	0.60 ± 0.12	0.63 ± 0.12	0.56 ± 0.12	0.08 ± 0.07	0.49 ± 0.14	0.12 ± 0.10	0.16 ± 0.09
EPA	0.808	0.385	0.79 ± 0.09	0.55 ± 0.12	0.63 ± 0.12	0.80 ± 0.09	0.45 ± 0.12	0.52 ± 0.12	0.37 ± 0.13	0.29 ± 0.11
DPA	0.728	0.620	0.74 ± 0.10	0.41 ± 0.12	0.57 ± 0.13	0.80 ± 0.09	0.43 ± 0.12	0.49 ± 0.13	0.36 ± 0.13	0.20 ± 0.10
DHA	0.897	0.375	0.84 ± 0.07	0.76 ± 0.09	0.66 ± 0.12	0.79 ± 0.09	0.34 ± 0.11	0.28 ± 0.11	0.52 ± 0.13	0.24 ± 0.10
FA_Bulk_	1.000	1.000	0.83 ± 0.07	0.68 ± 0.11	0.66 ± 0.11	0.80 ± 0.08	0.41 ± 0.11	0.42 ± 0.11	0.51 ± 0.12	0.35 ± 0.10

**Figure 3:**
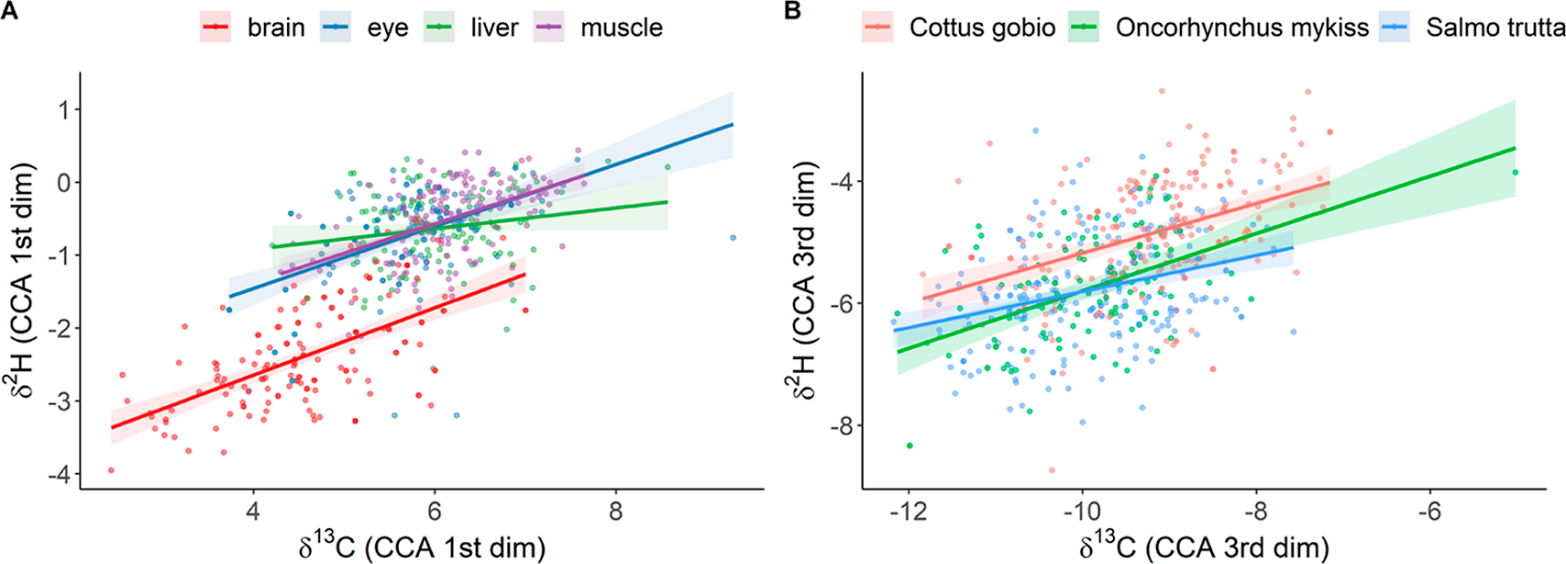
(A) Canonical correlation analysis (CCA) of tissue FA δ^2^H and δ^13^C data revealed significant isotopic separation of the brain from other tissue types in the first dimension. (B) Additionally, the third dimension revealed some minor differences between salmonids and bullheads.

Paired δ^2^H and δ^13^C-isotope data for 14:0, 16:0, 16:1, 18:0, 18:1, LIN, ALA, ARA, EPA, DPA, and DHA were measured in all samples and used for further investigation of sampling site specificity. HTA was measured in all brain samples and was considered when comparing brain samples. For further investigation of the effect of taxa, tissue type, and site on the variation of FA isotope data, CCA of δ^13^C and δ^2^H values of the eleven FA species was performed. The first seven dimensions of the CCA were significant (Wilks’ Lambda, *p* < 0.05). The first dimension had a canonical correlation of 0.721 (Wilks’ Lambda *F*-approx_12,14,141_ = 9.1, *p* < 0.001) and was primarily explained by the isotopic variation between the tissue types, particularly the brain (ANOVA Type II, tissue: *F*_3_ = 326.7, *p* < 0.001; site: *F*_15_ = 11.6, *p* < 0.001; taxa: *F*_2_ = 4.5, *p* = 0.011). Separation of brain δ values from other tissues was higher for *O. mykiss* and *S. trutta* than for *C. gobio* (Figure 3A) and was primarily influenced by δ^13^C_16:0_ (ccoef = −1.21), δ^13^C_EPA_ (ccoef = 0.83), δ^2^H_18:1_ (ccoef = 0.62), δ^2^H_ALA_ (ccoef = −0.47), δ^2^H_16:0_ (ccoef = −0.36), and δ^13^C_18:0_ (ccoef = 0.36). Similarly, the second dimension showed a separation of isotope values between eye and other tissues, particularly in *C. gobio*. The third dimension (canonical correlation 0.484; Wilks’ Lambda *F*-approx_8,13,415_ = 4.7, *p* < 0.001) was highly taxa-specific and separated between *C. gobio* and salmonids (Figure 3B), but fish liver from other tissues (ANOVA Type II, taxa: *F*_2_ = 79.7, *p* < 0.001; tissue: *F*_3_ = 50.7, *p* < 0.001; site: *F*_15_ = 17.6, *p* < 0.001) due to high canonical coefficients of δ^13^C_ALA_ (1.06), δ^13^C_DHA_ (−0.78), δ^13^C_14:0_ (0.68), δ^2^H_ALA_ (0.66), δ^2^H_18:0_ (0.63), and δ^2^H_DHA_ (−0.49). We thus concluded that FA isotope discrimination among the tissue types was too large to allow for pooling of the FA isotope data; subsequently, all further analyses were conducted by tissue type.

### Site Specificity of CSIA versus Bulk Tissue

**Figure 4:**
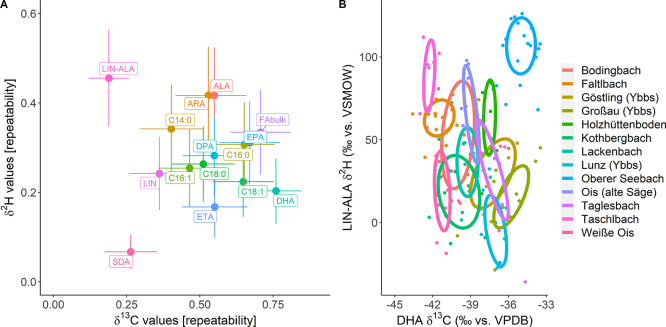
(A) Site-specific repeatability analysis, controlling for taxa, tissue type, and sampling year, revealed high site specificity for *n* – 3 LC-PUFA (e.g., DHA and EPA) ^13^C-CSIA, but not for ^2^H-CSIA, in which ALA and ARA showed higher specificity. (B) Plotting the isotopic difference between δ^2^H_ALA_ and δ^2^H_LIN_ against δ^13^C_DHA_ already revealed site-specific clusters in the fish muscle tissue (ellipses show 50% CI). More distinct site-specific separation was achieved when plotting the first three dimensions of a linear discrimination analysis, which can be seen in the interactive view of the online Supporting Information of this article (Figure S3: δ^2^H and δ^13^C values of 11 FA quantified in all samples; proportions of trace: LD1 = 48.2%, LD2 = 23.6%, LD3 = 7.8%).

Bootstrapping repeatability analysis was performed to assess to which extent the variability among isotopic data of a particular FA could be explained by the sampling site (Figure 2A). Repeatability varied among compounds depending on the tissue type (Table 2). In the case of δ^13^C, no individual FA compound provided significantly better site repeatability than FA_Bulk_ [mean: 74% ± 10]. δ^13^C_*n*–3 LC-PUFA_ values had similar site-specific repeatability as δ^13^C_FABulk_, particularly in the brain and muscle tissue, while the repeatability for *n* – 6 PUFA was poor across all tissue types. Among the non-essential (i.e., saturated and mono-unsaturated) FA, δ^13^C_16:0_ had the best site repeatability, especially for the muscle tissue [81.0% ± 10], which is higher than bulk sample repeatability, while δ^13^C_18:1_ showed high site repeatability in brain [74% ± 10] and eye tissue [71% ± 10]. In contrast, several FA showed better site-specific repeatability in their δ^2^H values compared to the δ^2^H-FA_Bulk_ [mean: 42% ± 11]. The δ^2^H_ALA_ values performed particularly well among all tissue types [mean: 54% ± 12], while site-specific repeatability for δ^2^H_LIN_ was high for the muscle tissue [59% ± 12]. δ^2^H_DHA_ showed some site-specific repeatability only for the liver tissue [52% ± 13], while those of 16:0 and 14:0 performed equally or better than FA_Bulk_ in the brain and eye tissue. The highest repeatability for any of the compound-specific δ^2^H values among all samples was achieved when using the difference between δ^2^H_LIN_ and δ^2^H_ALA_ (Figure 4A). Site-specific repeatability of the individual FA for both δ^2^H and δ^13^C did not change significantly when considering different sampling years.

Best-performing variables for site specificity (δ^13^C_DHA_ and δ^2^H_LIN-ALA_) revealed several site-specific clusters (Figure 4B). However, site-specific resolution was further improved by linear discrimination analysis based on a multivariate isotope approach (>2 FA). Highest site specificity was achieved for muscle samples, followed by eye, brain, and liver samples (Table 1). In total, in 141 of 148 fish considered (95%), and for 124 of the 148 fish when including bootstrapping cross-validation (84%), at least three out of the four tissue types were assigned correctly (Table 1). Site specificity accuracy was not improved by taxa-separated analysis but decreased when isotope data of different tissue types were combined.

Based on their close Mahalanobis distance in the δ^2^H_FA_ and δ^13^C, as well as their close geographical sampling locations (Figure 1), the fish samples from all sampling sites in Kothbergbach, Seebach, Weißer Ois, as well as Lunz and Lunz Großau were pooled to simplify further analysis. Data pooling further improved the site specificity of δ^2^H and δ^13^C (muscle: 94.6% [cv: 81.4%], eye: 94.3% [cv: 76.2%], brain: 95.4% [cv: 78.8%], liver: 95.4% [cv: 75.9%]). In linear discrimination analysis, the proportions of variation explained for the combined first three dimensions were >70% for all tissues (muscle: 48.2, 23.6, and 7.8%; eye: 40.2, 20.7, and 11.4%; brain: 54.1, 17.7, and 10.7%; liver: 54.1, 14.9, and 8.5%) and provided good visual discrimination of the site-specific clusters in a 3D plot (see online Supporting Information).

### Site-Specific Changes of FA Stable Isotope Values over Time

For evaluating the δ^2^H_FA_ and δ^13^C_FA_ values in the sedentary bullheads over time (site repeatability), we compared the δ^2^H_FA_ and δ^13^C_FA_ data of 2018 with those of 2016 from the same sampling sites. Both δ^2^H_FA_ and δ^13^C_FA_ values showed significant between-year isotopic differences in various tissues and locations (Table 3, Figure S3). In general, omega-6 PUFA frequently had larger isotopic differences than omega-3 PUFA. Saturated FA showed small changes in mean δ^13^C values. Changes in δ^2^H_18:0_ values were accompanied by an inverse proportional change in δ^2^H_LIN_ and δ^2^H_ARA_.

**Table 3:**
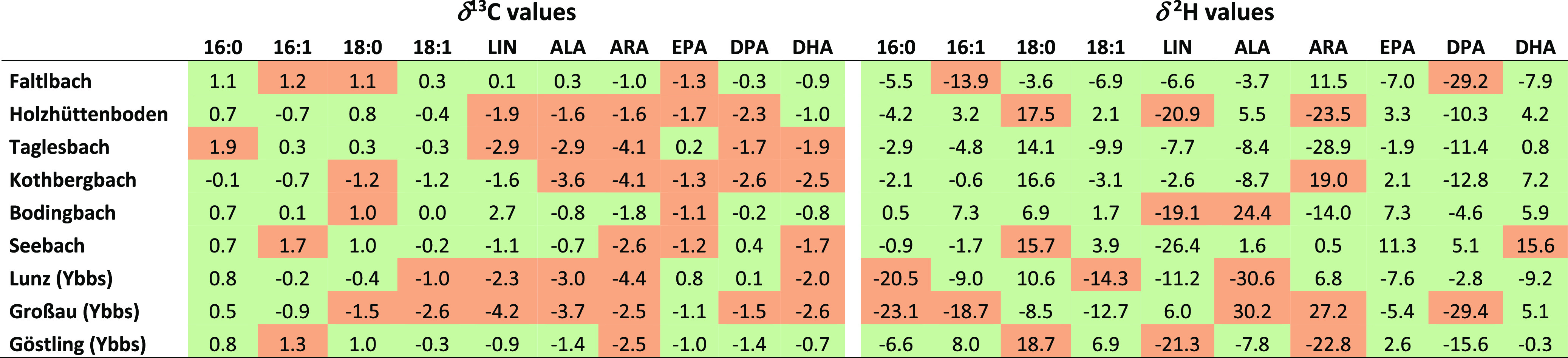
Mean Change of δ Values between 2016 and 2018^a^

a Red indicates statistically significant (*t*-test, *p* < 0.05) changes of δ values between both sampling time points.

Using *caret* (R package), a training data set (grand means of LDA) of bullhead samples from 2016 was generated to predict the sites of the bullhead samples from 2018. 34% of samples from 2018 were assigned to the correct location from the 15 sampling sites based on the isotopic composition of FA. This proportion increased to 54%, if accounting for assignments to a spatially close sampling location (< 1 km). In general, samples from tributaries at higher altitudes had a higher probability of being assigned to the correct location than samples from the further downstream sites of this study catchment (Table 4).

**Table 4:** Site Prediction of Samples from 2018 Based on the Training Set from 2016^a^

	Faltlbach	Holzhüttenboden	Taglesbach	Kothbergbach	Bodingbach	Seebach	Lunz (Ybbs)	Großau (Ybbs)	Göstling (Ybbs)
Faltlbach	17	2	0	2	2	0	0	0	0
Holzhüttenboden	1	4	6	0	7	0	2	6	0
Taglesbach	0	8	1	1	9	0	0	0	0
Kothbergbach	10	0	1	9	2	0	0	0	0
Bodingbach	1	0	3	1	11	0	3	1	0
Seebach	0	6	0	0	1	10	0	1	1
Lunz (Ybbs)	0	7	1	1	6	0	6	1	0
Großau (Ybbs)	0	3	0	1	3	0	7	2	0
Göstling (Ybbs)	0	2	4	0	2	1	4	3	2

a 34% of all samples from 2018 were assigned to the correct location based on the isotopic composition of FA.

## Discussion

The δ^2^H and δ^13^C analyses of individual FA in fish tissues provided more accurate information on fish site specificity than their bulk FA stable isotope compositions. Our compound-specific stable isotope analysis of FA showed that within a short distance (e.g., <5 km) between sampling sites of these streams, fish tissues had unique δ^2^H_FA_ and δ^13^C_FA_ values that allowed assignment of fish origin at a small spatial scale.

The δ^13^C_FA_ values of fish were more negative at higher altitude sites, and the δ^13^C values of individual FA were highly correlated with each other.

In contrast to changes in δ^13^C values with altitude, no such effect was observed for δ^2^H and the variation among FA was not explained by a calculated δ^2^H_Bulk_ value. It is possible that different physiological requirements for fish at the higher and lower altitude sampling sites altered fish metabolism, which is already known to change δ^2^H values of long-chain (>18) PUFA.^40^ Thus, δ^2^H values of individual FA, for example, highly required EPA and DHA, likely change because of FA bioconversion processes in case of insufficient dietary supply.^30^ This is also reflected by the fact that the difference in isotopic values δ^2^H_LIN-ALA_ showed the highest site specificity for δ^2^H_FA_ values. Consumers are not able to synthesize LIN or ALA *de novo*,^41^ and thus, the isotope values likely reflect those of the base of the food web, whose structure can also show site-specific differences. This suggests that δ^2^H_FA_ showed less site specificity compared to δ^13^C_FA_, although δ^2^H_FA_ values can reveal fine-scale structures in small geographical areas where larger-scale δ^2^H patterns of water are absent.

The *n* – 3 PUFA DHA, EPA, and ALA, as well as the saturated FA 16:0 and the mono-unsaturated FA 18:1 had high predictive δ^13^C values of sampling site specificity. It is likely that these FA are important for maintaining tissue function, are required in high quantities, and are retained in storage and functional membrane lipids,^42^ thereby keeping the dietary isotopic values. While 16:0 and 18:1 are non-essential FA and thus might be also synthesized *de novo* by consumers, they are also available in high levels from the diet (e.g., invertebrates), and hence, their isotopic values are also more likely to reflect local food sources. Interestingly, LIN dual-isotope data did not correlate with the sampling site, in any tissue, despite being an essential FA. LIN is the precursor of the *n* – 6 LC-PUFA ARA, an important tissue FA regulating inflammatory processes in vertebrates.^43^ It thus might be used mainly for energy production, as the precursor for elongation to ARA or to regulate *n* – 3 to *n* – 6 bioconversion rates because *n* – 3 and *n* – 6 PUFA use the same bioconverting enzymes.^44^ All the above involve several biochemical conversion processes that might induce significant isotopic fractionation of LIN,thereby blurring site-specific isotopic information of this FA.

In addition to sampling site-specific FA isotope variation, tissue and species type also differed isotopically. For example, brain FA had comparatively higher δ^13^C_SFA_ and δ^13^C_MUFA_ values and lower δ^2^H_SFA_ and δ^2^H_MUFA_ values compared to muscle or liver tissues, which may be due to the unique metabolism of the brain; (1) relying more on carbohydrates than fat for ATP production;^45^ (2) being separated by the blood–brain barrier restricting the exchange of molecules between blood and the rest of the body, and/or (3) relying on its own FA synthesizing complex, for example, for building very long-chain FA that isolate axons as part of the myelin sheet.^46^ Fatty acids are thereby synthesized, particularly during growth phases of an individual, using ketone bodies (e.g., ß-hydroxybutyrate and acetoacetate) as sources, in contrast to acetate, which is used for FA synthesis in the liver.^46^ These different biochemical synthesizing pathways might therefore explain these isotopic differences in saturated and mono-unsaturated FA between brain and the other tissues.

When using δ^2^H_FA_ and δ^13^C_FA_ values as site-specific markers in canonical variation analysis (Table 1), it was possible to classify fish samples with over 90% accuracy to their spatial origin, and over 70% when performing bootstrapping analysis, which was considerably better than concomitant bulk FA isotope values (∼40% accuracy). Isotopic overlap and wrongly assigned samples could be due to the proximity of some sampling sites (e.g., between the contributary stream Bodingbach and Lunz: ∼1 km; and between Lackenbach and Alte Säge/Ois: ∼300 m) despite being supplied by different water sources that might share similar local physiological and isotopic properties, such as evaporation, or food sources leading to similar isotopic discrimination of FA in fish tissues. Also, fish were more likely to have moved between these sites, thereby averaging the signal of their tissues. Therefore, our data support the use of CSIA of FA as site-specific markers, although their application across larger spatial areas with strong H-isotope gradients (isoscapes) remains to be investigated, as well as the implication’s limitation relating the natural range of isotopic variation to the spatial resolution.

The δ^13^C_FA_ values differed slightly, but significantly, between the 2016 and 2018 samples, revealing that isotopic patterns vary over time and that samples from different time points cannot be compared across periods of time (i.e., across several seasons). Nevertheless, this study provides compound-specific isotopic evidence that the fish origin within this stream network (i.e., site specificity) could be ensured with 54% accuracy across the two years, which was more precise than using only bulk stable isotopes for site specificity within a single season. Compound-specific isotope values generally have higher variation than bulk stable isotope values on larger isoscapes,^1,19,47,48^ implying that local food web and community structures as well as physiological processes have a higher influence on stable isotope composition of FA than meteorologic isotopic fractionation. Naturally occurring inter-annual δ^2^H and δ^13^C variations at the molecular level might be more sensitive to small environmental changes, which are largely smoothed out when analyzing bulk tissues with longer turnover times (e.g., muscle tissues and brain), while changes are quickly reflected in high-turnover tissue, such as liver. In this case, tissue FA isotopic differences could potentially be used to improve tracking migration of individual fish. This finding might provide a meaningful estimate of the fish origin, especially if considering larger-scale catchments. However, the natural range of FA isotopic variation needs to be better defined by location and over time.

Unfortunately, there are no movement data for this retrospective assessment, however, we found relatively small isotopic differences between salmonids and bullheads compared to the overall isotopic variations. Therefore, these differences are possibly attributed to different feeding behaviors rather than different movement patterns, which remains to be clarified in future studies.

Our findings suggest that the FA isotope composition in fish tissues can help better identify the provenance of fish samples, even at small geographical scales. We summarize the following aspects as recommendations for future research:

(1) No clear spatio-temporal patterns in the FA isotopic composition can be applied for all cases. Resident fish grown in enclosures, ponds, or with limited capacity for migration need to be sampled to assess the isotope variation within the area of interest before evaluating samples of unknown origin (“known-diet source approach”). Controlled captive studies using isotope gradients in the sources (water and diet) and different levels of dietary quality to quantify the mechanisms that drive the isotopic flow and dynamics in FA are needed to understand the strengths and caveats of these CSIA to better assign the origin of fish in aquatic ecosystems.
(2) One technical aspect for the use of this isotope tool is how researchers determine the FA-CSIA baseline to distinguish between sites or areas of interest. Whether a resident fish species to discriminate among sites or the use of a lower trophic level organism, such as macroinvertebrates or periphyton, dependent on the FA type (essential vs non-essential) and time of sampling.
(3) As with bulk tissue isotope methods, more detailed knowledge is warranted for specific tissue/s FA isotope turnover times and integration period (muscle vs brain; summer vs winter; “ecophysiological approach”).
(4) The use of multi-isotope molecular approaches is recommended to improve data and spatial assignment resolution in other environments. The current study focused on a small sub-alpine stream catchment, and the applicability of this methodological approach to other stream systems or by using anadromous species remains to be elucidated. Well-known and predictable hydrogen isotope gradients in water based on latitude or marine versus freshwater studies may enhance the potential of using FA H-isotopes for this method. The dual-isotope approach at the FA level makes site discrimination easier without increasing time on sample treatment; however, it will require two separate CSIAs.
(5) Statistical approach of large data sets: multivariate data analysis including several FA is recommended for a priori site specificity, but refined protocols with a wider selection of FA can be investigated. As most of the variation among the data originates from the FA compound, CCA can be a useful tool to assess the influence of other factors than location and to decide for which samples a pooled analysis can or should not be performed.
(6) Finally, the addition of CSIA of amino acids might add another dimension to further increase accuracy and may be included in future studies.

In conclusion, the combination of δ^13^C_FA_ and δ^2^H_FA_ values provided higher spatial resolution assignment of fish origin in this stream network than bulk FA isotopic analysis. The isotopic patterns resulted from a combination of physicochemical processes that had a strong influence on δ^13^C values, and possible ecophysiological processes with effects mainly on δ^2^H values of individual FA. The compound-specific isotopic composition of the samples gained from individuals thus reflects the integrated effect of element isoscapes, diet, and metabolism. The latter has the potential to induce significant metabolic fractionation, especially for δ^2^H values, disrupting the preservation of isotopic values of the diet. In that sense, this method primarily compares individuals regarding their similarity in life history, among which residency and feeding at a particular location is an important factor, without requiring a baseline or knowledge about all potential food sources. How topological and ecophysiological properties influence the isotopic patterns of FA, how isotopic patterns of FA change in response to different trophic levels or at larger spatial scales, and if non-migrant fish require to establish local isotopic patterns remain questions for future migration ecology research. This introduced method enhances our capacity to trace foraging and migration of fishes where other telemetric methods are not applicable, and it can provide vital information about habitat degradation of endangered species and spread of an invasive species.^49^ Specific examples of such application are to determine the impact of vertical barriers on migration of small fish species (e.g., from genus *Cottus* or *Gobio*). The CSIA of FA of eggs and early life stages recovered at the spawning grounds can provide important information on foraging grounds of adults^50^ and lipid-rich tissues with slow turnover (e.g., brain and eye lens) could also help assess the hatchery or wild origin of fish stocked to the natural systems.^51^

## Supporting Information Available

Mean fatty acid bulk δ^13^C and δ^2^H values and standard deviation at individual sampling sites (2016); changes in fish fatty acid isotope values for (A) δ^13^C and (B) δ^2^H at nine different sampling sites between 2016 and 2018; and LDA of δ^2^H and δ^13^C values of 11 FA quantified in all samples (PDF)
Interactive 3D plot of the first three dimensions of the LDA site-specificity plot (ZIP)

## References

[ref1] Hobson K. A. Tracing Origins and Migration of Wildlife Using Stable Isotopes: A Review. Oecologia 1999, 120, 314–326. 10.1007/s004420050865. 28308009

[ref2] Tigano A.; Friesen V. L. Genomics of Local Adaptation with Gene Flow. Mol. Ecol. 2016, 25, 2144–2164. 10.1111/mec.13606. 26946320

[ref3] Wilkes M. A.; Webb J. A.; Pompeu P. S.; Silva L. G. M.; Vowles A. S.; Baker C. F.; Franklin P.; Link O.; Habit E.; Kemp P. S. Not Just a Migration Problem: Metapopulations, Habitat Shifts, and Gene Flow Are Also Important for Fishway Science and Management. River Res. Appl. 2019, 35, 1688–1696. 10.1002/rra.3320.

[ref4] Samways K. M.; Soto D. X.; Cunjak R. A. Aquatic Food-Web Dynamics Following Incorporation of Nutrients Derived from Atlantic Anadromous Fishes. J. Fish. Biol. 2018, 92, 399–419. 10.1111/jfb.13519. 29235101

[ref5] Burtner A. M.; McIntyre P. B.; Allan J. D.; Kashian D. R. The Influence of Land Use and Potamodromous Fish on Ecosystem Function in Lake Superior Tributaries. J. Great Lake. Res. 2011, 37, 521–527. 10.1016/j.jglr.2011.05.014.

[ref6] Hassan M. A.; Gottesfeld A. S.; Montgomery D. R.; Tunnicliffe J. F.; Clarke G. K. C.; Wynn G.; Jones-Cox H.; Poirier R.; MacIsaac E.; Herunter H.; Macdonald S. J. Salmon-Driven Bed Load Transport and Bed Morphology in Mountain Streams. Geophys. Res. Lett. 2008, 35, (4), . DOI: 10.1029/2007GL032997.

[ref7] van Treeck R.; Radinger J.; Noble R. A. A.; Geiger F.; Wolter C. The European Fish Hazard Index—An assessment tool for screening hazard of hydropower plants for fish. Sustain. Energy Technol. Assessments 2021, 43, 100903 10.1016/j.seta.2020.100903.

[ref8] Chambers P. A.; Vis C.; Brua R. B.; Guy M.; Culp J. M.; Benoy G. A. Eutrophication of Agricultural Streams: Defining Nutrient Concentrations to Protect Ecological Condition. Water Sci. Technol. 2008, 58, 2203–2210. 10.2166/wst.2008.815. 19092197

[ref9] Dodds W. K.; Smith V. H. Nitrogen, Phosphorus, and Eutrophication in Streams. Inland Waters 2016, 6, 155–164. 10.5268/IW-6.2.909.

[ref10] Myers J. P.; Morrison R. I. G.; Antas P. Z.; Harrington B. A.; Lovejoy T. E.; Sallaberry M.; Senner S. E.; Tarak A. Conservation Strategy for Migratory Species. Am. Sci. 1987, 75, 19–26.

[ref11] Olden J. D.; Kennard M. J.; Leprieur F.; Tedesco P. A.; Winemiller K. O.; García-Berthou E. Conservation Biogeography of Freshwater Fishes: Recent Progress and Future Challenges. Divers. Distrib. 2010, 16, 496–513. 10.1111/j.1472-4642.2010.00655.x.

[ref12] Zentner D. L.; Wolf S. L.; Brewer S. K.; Shoup D. E. A Review of Factors Affecting PIT Tag Detection Using Mobile Arrays and Use of Mobile Antennas to Detect PIT-Tagged Suckers in a Wadeable Ozark Stream. N. Am. J. Fish. Manag. 2021, 41, 697–710. 10.1002/nafm.10578.

[ref13] Brownscombe J. W.; Lédée E. J. I.; Raby G. D.; Struthers D. P.; Gutowsky L. F. G.; Nguyen V. M.; Young N.; Stokesbury M. J. W.; Holbrook C. M.; Brenden T. O.; Vandergoot C. S.; Murchie K. J.; Whoriskey K.; Mills Flemming J.; Kessel S. T.; Krueger C. C.; Cooke S. J. Conducting and Interpreting Fish Telemetry Studies: Considerations for Researchers and Resource Managers. Rev. Fish Biol. Fish. 2019, 29, 369–400. 10.1007/s11160-019-09560-4.

[ref14] Hobson K. A.; Norris D. R.; Kardynal K. J.; Yohannes E. Animal Migration. In Tracking Animal Migration with Stable Isotopes, 2nd ed.; Hobson K. A.; Wassenaar L. I., Eds.; Academic Press, 2019, pp 1–23.

[ref15] Fry B. Stable Isotope Ecology; Springer: New York, 2006.

[ref16] Hobson K.; Wassenaar L. Tracking Animal Migration with Stable Isotopes, 2nd ed.; Academic Press, 2018.

[ref17] Hobson K. A. Application of Isotopic Methods to Tracking Animal Movements. In Tracking Animal Migration with Stable Isotopes, 2nd ed.; Hobson K. A.; Wassenaar L. I., Ed.; Academic Press, 2019, pp 85–115.

[ref18] Soto D. X.; Hobson K. A.; Wassenaar L. I. Using Hydrogen Isotopes of Freshwater Fish Tissue as a Tracer of Provenance. Ecol. Evol. 2016, 6, 7776–7782. 10.1002/ece3.2519. 30128127 PMC6093159

[ref19] Winter E. R.; Hindes A. M.; Lane S.; Britton J. R. Dual-Isotope Isoscapes for Predicting the Scale of Fish Movements in Lowland Rivers. Ecosphere 2021, 12, e03456 10.1002/ecs2.3456.

[ref20] Rachel B.-J.; Pearson T. E.; Ramos F. C.; Grimes C. B.; Bruce MacFarlane R. Tracking Natal Origins of Salmon Using Isotopes, Otoliths, and Landscape Geology. Limnol. Oceanogr. 2008, 53, 1633–1642. 10.4319/lo.2008.53.4.1633.

[ref21] McMahon K. W.; Newsome S. D. Amino Acid Isotope Analysis. In Tracking Animal Migration with Stable Isotopes, 2nd ed.; Hobson K. A.; Wassenaar L. I., Ed.; Academic Press, 2019, pp 173–190.

[ref22] Gómez C.; Larsen T.; Popp B.; Hobson K. A.; Cadena C. D. Assessing Seasonal Changes in Animal Diets with Stable-Isotope Analysis of Amino Acids: A Migratory Boreal Songbird Switches Diet over Its Annual Cycle. Oecologia 2018, 187, 1–13. 10.1007/s00442-018-4113-7. 29564539

[ref23] Pilecky M.; Winter K.; Wassenaar L. I.; Kainz M. J. Compound-Specific Stable Hydrogen Isotope (Δ2H) Analyses of Fatty Acids: A New Method and Perspectives for Trophic and Movement Ecology. Rapid Commun. Mass Spectrom. 2021, 35, e9135 10.1002/rcm.9135. 34080229 PMC11478936

[ref24] Stewart G. R.; Turnbull M. H.; Schmidt S.; Erskine P. D. 13C Natural Abundance in Plant Communities Along a Rainfall Gradient: A Biological Integrator of Water Availability. Funct. Plant Biol. 1995, 22, 51–55. 10.1071/pp9950051.

[ref25] Männel T. T.; Auerswald K.; Schnyder H. Altitudinal Gradients of Grassland Carbon and Nitrogen Isotope Composition Are Recorded in the Hair of Grazers. Global Ecol. Biogeogr. 2007, 16, 583–592. 10.1111/j.1466-8238.2007.00322.x.

[ref26] Hobson K. A.; Wassenaar L. I.; Milá B.; Lovette I.; Dingle C.; Smith T. B. Stable Isotopes as Indicators of Altitudinal Distributions and Movements in an Ecuadorean Hummingbird Community. Oecologia 2003, 136, 302–308. 10.1007/s00442-003-1271-y. 12756525

[ref27] Gong X.; Xu Z.; Peng Q.; Tian Y.; Hu Y.; Li Z.; Hao T. Spatial patterns of leaf δ^13^C and δ^15^N of aquatic macrophytes in the arid zone of northwestern China. Ecol. Evol. 2021, 11, 3110–3119. 10.1002/ece3.7257. 33841771 PMC8019054

[ref28] Waigwa A. N.; Mwangi B. N.; Gituru R. W.; Omengo F.; Zhou Y.; Wang Q. Altitudinal Variation of Leaf Carbon Isotope for Dendrosenecio Keniensis and Lobelia Gregoriana in Mount Kenya Alpine Zone. Biotropica 2021, 53, 1394–1405. 10.1111/btp.12990.

[ref29] Campeau A.; Wallin M. B.; Giesler R.; Löfgren S.; Mörth C.-M.; Schiff S.; Venkiteswaran J. J.; Bishop K. Multiple Sources and Sinks of Dissolved Inorganic Carbon across Swedish Streams, Refocusing the Lens of Stable C Isotopes. Sci. Rep. 2017, 7, 9158 10.1038/s41598-017-09049-9. 28831088 PMC5567220

[ref30] Guo F.; Ebm N.; Bunn S. E.; Brett M. T.; Hager H.; Kainz M. J. Longitudinal Variation in the Nutritional Quality of Basal Food Sources and Its Effect on Invertebrates and Fish in Subalpine Rivers. J. Anim. Ecol. 2021, 90, 2678 10.1111/1365-2656.13574. 34358339

[ref31] Fischer S.; Kummer H. Effects of Residual Flow and Habitat Fragmentation on Distribution and Movement of Bullhead (Cottus Gobio L.) in an Alpine Stream. Hydrobiologia 2000, 422, 305–317. 10.1007/978-94-011-4164-2_25.

[ref32] Nevoux M.; Finstad B.; Davidsen J. G.; Finlay R.; Josset Q.; Poole R.; Höjesjö J.; Aarestrup K.; Persson L.; Tolvanen O.; Jonsson B. Environmental Influences on Life History Strategies in Partially Anadromous Brown Trout (Salmo Trutta, Salmonidae). Fish Fish. 2019, 20, 1051–1082. 10.1111/faf.12396.

[ref33] Slavík O.; Horký P. Home Range Size Decreases with Increasing Site Fidelity in High-Density Subpopulations of Brown Trout. Ethol. Ecol. Evol. 2019, 31, 421–434. 10.1080/03949370.2019.1624277.

[ref34] Guo F.; Bunn S. E.; Brett M. T.; Fry B.; Hager H.; Ouyang X.; Kainz M. J. Feeding Strategies for the Acquisition of High-quality Food Sources in Stream Macroinvertebrates: Collecting, Integrating, and Mixed Feeding. Limnol. Oceanogr. 2018, 63, 1964–1978. 10.1002/lno.10818. 30555183 PMC6283091

[ref35] Ebm N.; Guo F.; Brett M. T.; Bunn S. E.; Kainz M. J. Polyunsaturated Fatty Acids in Fish Tissues More Closely Resemble Algal than Terrestrial Diet Sources. Hydrobiologia 2021, 848, 371–383. 10.1007/s10750-020-04445-1. 33343020 PMC7738338

[ref36] Heissenberger M.; Watzke J.; Kainz M. J. Effect of Nutrition on Fatty Acid Profiles of Riverine, Lacustrine, and Aquaculture-Raised Salmonids of Pre-Alpine Habitats. Hydrobiologia 2010, 650, 243–254. 10.1007/s10750-010-0266-z.

[ref37] Holm S. A Simple Sequentially Rejective Multiple Test Procedure. Scandinavian Journal of Statistics; JSTOR, 1979, Vol. 6, pp 65–70.

[ref38] Nakagawa S.; Schielzeth H. Repeatability for Gaussian and Non-Gaussian Data: A Practical Guide for Biologists. Biol. Rev. Cambridge Philos. Soc. 2010, 85, 935–956. 10.1111/j.1469-185X.2010.00141.x. 20569253

[ref39] Searle S. R.; Speed F. M.; Milliken G. A. Population Marginal Means in the Linear Model: An Alternative to Least Squares Means. Am. Statistician 1980, 34, 216–221. 10.1080/00031305.1980.10483031.

[ref40] Pilecky M.; Kämmer S. K.; Mathieu-Resuge M.; Wassenaar L. I.; Taipale S. J.; Martin-Creuzburg D.; Kainz M. J. Hydrogen isotopes (δ^2^H) of polyunsaturated fatty acids track bioconversion by zooplankton. Funct. Ecol. 2021, 36, 538–549. 10.1111/1365-2435.13981.

[ref41] Cook H.; McMaster C. Chapter 7 Fatty Acid Desaturation and Chain Elongation in Eukaryotes. N. Compr. Biochem. 2002, 36, 181–204. 10.1016/S0167-7306(02)36009-5.

[ref42] Pilecky M.; Závorka L.; Arts M. T.; Kainz M. J. Omega-3 PUFA profoundly affect neural, physiological, and behavioural competences—implications for systemic changes in trophic interactions. Biol. Rev. 2021, 96, 2127–2145. 10.1111/brv.12747. 34018324

[ref43] Innes J. K.; Calder P. C. Omega-6 Fatty Acids and Inflammation. Prostagl. Leukot. Essent. Fat. Acids 2018, 132, 41–48. 10.1016/j.plefa.2018.03.004. 29610056

[ref44] Voss A.; Reinhart M.; Sankarappa S.; Sprecher H. The Metabolism of 7,10,13,16,19-Docosapentaenoic Acid to 4,7,10,13,16,19-Docosahexaenoic Acid in Rat Liver Is Independent of a 4-Desaturase. J. Biol. Chem. 1991, 266, 19995–20000. 10.1016/S0021-9258(18)54882-1. 1834642

[ref45] Soengas J. L.; Aldegunde M. Energy Metabolism of Fish Brain. Comp. Biochem. Physiol B Comp. Biochem. Mol. Biol. 2002, 131, 271–296. 10.1016/S1096-4959(02)00022-2. 11959012

[ref46] Nehlig A. Brain Uptake and Metabolism of Ketone Bodies in Animal Models. Prostagl. Leukot. Essent. Fat. Acids 2004, 70, 265–275. 10.1016/j.plefa.2003.07.006. 14769485

[ref47] Hobson K. A.; Soto D. X.; Paulson D. R.; Wassenaar L. I.; Matthews J. H. A dragonfly (δ^2^H) isoscape for North America: a new tool for determining natal origins of migratory aquatic emergent insects. Methods Ecol. Evol. 2012, 3, 766–772. 10.1111/j.2041-210X.2012.00202.x.

[ref48] Radabaugh K. R.; Hollander D. J.; Peebles E. B. Seasonal δ^13^C and δ^15^N isoscapes of fish populations along a continental shelf trophic gradient. Cont. Shelf Res. 2013, 68, 112–122. 10.1016/j.csr.2013.08.010.

[ref49] Cunjak R. A.; Roussel J.-M.; Gray M. A.; Dietrich J. P.; Cartwright D. F.; Munkittrick K. R.; Jardine T. D. Using Stable Isotope Analysis with Telemetry or Mark-Recapture Data to Identify Fish Movement and Foraging. Oecologia 2005, 144, 636–646. 10.1007/s00442-005-0101-9. 15959824

[ref50] Charles K.; Roussel J.-M.; Cunjak R. A. Estimating the Contribution of Sympatric Anadromous and Freshwater Resident Brown Trout to Juvenile Production. Mar. Freshw. Res. 2004, 55, 185 10.1071/MF03173.

[ref51] Bell-Tilcock M.; Jeffres C. A.; Rypel A. L.; Sommer T. R.; Katz J. V. E.; Whitman G.; Johnson R. C. Advancing Diet Reconstruction in Fish Eye Lenses. Methods Ecol. Evol. 2021, 12, 449–457. 10.1111/2041-210X.13543.

