# Supplementary material for: Assessment of Compound-Specific Fatty Acid δ13C and δ2H Values to Track Fish Mobility in a Small Sub-alpine Catchment
Source: Environ Sci Technol. 2022 Jul 21;56(15):11051–60. doi: 10.1021/acs.est.2c02089 (PMC9352314; doi:10.1021/acs.est.2c02089)
Supplement: Supplementary file 1 — es2c02089_si_001.pdf [file es2c02089_si_001.pdf]

Assessment of compound-specific fatty acid δ^{13}C and δ^{2}H values to track fish mobility in a small subalpine catchment

Matthias Pilecky[1,2,*], Libor Závorka[1], David X. Soto[3], Fen Guo[4], Leonard I. Wassenaar[1,2,5], and Martin J. Kainz[1,2]

Supplementary Information

1) WasserCluster Lunz – Biologische Station, Inter-University Center for Aquatic Ecosystem Research, Dr. Carl-Kupelwieser Promenade 5, 3293 Lunz am See, Austria
2) Donau-Universität Krems, Department for Biomedical Research, Dr. Karl-Dorrek-Straße 30, 3500 Krems, Austria
3) International Atomic Energy Agency, Isotope Hydrology Section, Vienna International Centre, A-1400, Vienna, Austria
4) Guangdong Provincial Key Laboratory of Water Quality Improvement and Ecological Restoration for Watersheds, Institute of Environmental and Ecological Engineering, Guangdong University of Technology, Guangzhou, 511458, China
5) University of Saskatchewan, Dept. of Geological Science, 114 Science Place, Saskatoon, SK S7N 5E2, Canada

*) corresponding author

LZ: libor.zavorka@wcl.ac.at (ORCID: 0000-0002-0489-3681)

MP: matthias.pilecky@donau-uni.ac.at (0000-0002-3404-5923**)**

MJK: martin.kainz@donau-uni.ac.at (ORCID: 0000-0002-2388-1504)

FG: fen.guo@gdut.edu.cn (ORCID: 0000-0002-4976-5456)

LIW: len.wassenaar@usask.ca (ORCID: 0000-0001-5532-0771)

DXS: d.soto@iaea.org (ORCID: 0000-0003.4210-293X)

Table S1. Mean fatty acid bulk $\delta^{13}C$ and $\delta^{2}H$ values and standard deviation at individual sampling sites (2016).

			$\delta^{13}C_{FAbulk}$ vs. VPDB				$\delta^{2}H_{FAbulk}$ vs. VSMOW			
**Site**	**Taxa**	**n**	**brain**	**eye**	**liver**	**muscle**	**brain**	**eye**	**liver**	**muscle**
**Bodingbach**	*C. gobio*	3	-37.1 ± 0.3	-36.6 ± 0.0	-37.9 ± 1.0	-39.0 ± 0.9	-290.6 ± 1.4	-265.6 ± 0.0	-277.0 ± 21.7	-278.8 ± 22.9
	O. mykiss	3	-35.6 ± 0.2	-37.4 ± 1.5	-37.8 ± 1.4	-39.3 ± 0.8	-294.0 ± 10.4	-283.4 ± 12.5	-270.9 ± 15.7	-277.6 ± 13.5
	S. trutta	3	-37.1 ± 1.0	-37.7 ± 0.0	-38.5 ± 0.8	-38.4 ± 2.2	-306.1 ± 2.9	-290.7 ± 0.0	-276.3 ± 14.6	-290.0 ± 16.9
**Faltlbach**	*C. gobio*	13	-39.6 ± 0.8	-38.9 ± 1.9	-39.2 ± 1.5	-40.5 ± 1.2	-272.8 ± 5.8	-247.4 ± 15.3	-237.2 ± 10.1	-257.4 ± 11.9
**Kothbergbach (oben)**	*C. gobio*	4	-37.9 ± 0.9	-38.0 ± 0.0	-37.7 ± 1.0	-38.2 ± 1.6	-278.9 ± 4.9	-256.8 ± 0.0	-231.8 ± 4.1	-249.1 ± 2.3
	O. mykiss	1	-35.8 ± NA	-37.4 ± NA	-37.1 ± NA	-38.3 ± NA	-300.1 ± NA	-265.3 ± NA	-269.3 ± NA	-272.6 ± NA
	S. trutta	1	-34.3 ± NA	-37.4 ± NA	-37.2 ± NA	-37.8 ± NA	-285.3 ± NA	-258.2 ± NA	-243.2 ± NA	-256.4 ± NA
**Kothbergbach (unten)**	*C. gobio*	1	-38.1 ± NA	-39.8 ± NA	-38.4 ± NA	-38.4 ± NA	-288.1 ± NA	-258.5 ± NA	-251.1 ± NA	-264.5 ± NA
	S. trutta	5	-36.2 ± 0.7	-37.5 ± 1.7	-38.1 ± 1.1	-39.0 ± 1.3	-299.2 ± 8.0	-292.5 ± 8.7	-268.8 ± 7.9	-277.9 ± 5.3
**Lackenbach**	*C. gobio*	3	-37.6 ± NA	-37.8 ± NA	-38.0 ± NA	-38.6 ± 1.0	-290.9 ± NA	-274.5 ± NA	-255.7 ± NA	-282.6 ± 8.4
	O. mykiss	8	-36.3 ± 0.4	-38.1 ± 1.0	-37.6 ± 0.3	-38.8 ± 0.8	-297.0 ± 8.0	-275.6 ± 5.4	-272.7 ± 3.7	-282.5 ± 4.1
	S. trutta	1	-37.6 ± NA	-37.8 ± NA	NA	-38.0 ± NA	-290.9 ± NA	-274.5 ± NA	NA	-282.8 ± NA
**Oberer Seebach (Lend)**	*C. gobio*	3	-35.1 ± 1.0	-35.3 ± 0.9	-35.4 ± 0.2	-35.6 ± 0.5	-282.2 ± 17.9	-286.8 ± 0.3	-272.2 ± 9.9	-273.8 ± 13.1
	S. trutta	7	-34.0 ± 1.0	-36.0 ± 0.8	-35.6 ± 1.2	-36.3 ± 0.9	-295.8 ± 3.8	-281.6 ± 11.6	-264.9 ± 17.6	-275.1 ± 6.9
**Oberer Seebach (Ritrodat)**	*C. gobio*	5	-33.6 ± 0.4	-35.5 ± 1.6	-34.6 ± 1.2	-34.9 ± 1.4	-282.7 ± 12.0	-256.4 ± 16.0	-267.7 ± 7.5	-268.9 ± 9.1
	S. trutta	6	-32.7 ± 0.6	-34.9 ± 0.8	-34.9 ± 1.1	-35.4 ± 0.7	-296.3 ± 4.5	-277.0 ± 5.5	-267.1 ± 5.3	-278.3 ± 3.4
**Ois (alte Säge)**	*O. mykiss*	5	-36.8 ± 0.8	-39.0 ± 0.4	NA	-38.8 ± 0.3	-293.9 ± 6.2	-290.5 ± 5.7	NA	-284.7 ± 13.4
	S. trutta	5	-36.6 ± 1.7	-38.6 ± 1.5	NA	-38.8 ± 1.2	-289.8 ± 12.8	-284.1 ± 7.2	NA	-283.3 ± 7.1
**Ois (Holzhüttenboden)**	*C. gobio*	8	-35.7 ± 0.3	-35.5 ± 0.6	-36.6 ± 1.1	-37.0 ± 0.8	-282.2 ± 6.0	-269.2 ± 2.1	-261.7 ± 13.9	-278.3 ± 6.9
	S. trutta	3	-35.0 ± 0.4	-37.4 ± 1.5	-37.1 ± NA	-37.6 ± NA	-298.7 ± 3.9	-282.7 ± 0.4	-280.7 ± NA	-287.0 ± NA
**Tagles (unten)**	*C. gobio*	6	-36.1 ± 1.0	-37.6 ± 1.0	-37.2 ± 1.2	-37.4 ± 1.0	-292.0 ± 5.8	-280.2 ± 0.2	-283.6 ± 9.1	-284.1 ± 4.0
	S. trutta	6	-34.6 ± 0.7	-37.9 ± 2.1	-36.5 ± 1.3	-36.8 ± 1.0	-301.6 ± 6.0	-292.2 ± 3.7	-287.8 ± 5.7	-290.2 ± 4.0
**Taschlbach**	*S. trutta*	7	-39.6 ± 0.3	-41.9 ± 1.5	-40.1 ± 1.0	-42.0 ± 0.6	-301.3 ± 6.6	-280.2 ± 10.4	-254.2 ± 7.6	-278.7 ± 7.9
**Weiße Ois (Rehberghütte)**	*S. trutta*	16	-38.8 ± 0.9	-40.6 ± 0.9	-39.8 ± 1.4	-41.5 ± 1.1	-287.3 ± 5.7	-264.9 ± 3.4	-259.3 ± 10.9	-284.4 ± 6.2
**Weiße Ois (Faltl)**	*S. trutta*	2	-37.6 ± 1.8	-41.4 ± 2.8	-38.7 ± 2.3	-40.6 ± 2.9	-294.0 ± 4.2	-271.5 ± 0.3	-255.9 ± 7.3	-258.6 ± 14.9
**Ybbs (Göstling, Lagerhaus)**	*C. gobio*	5	-35.3 ± 0.7	-35.2 ± 0.9	-36.3 ± 1.6	-36.8 ± 1.3	-289.5 ± 4.4	-267.7 ± 8.5	-271.3 ± 8.3	-272.6 ± 14.4
	O. mykiss	6	-35.1 ± 0.6	-36.9 ± 1.1	-36.8 ± 0.6	-37.5 ± 0.9	-305.1 ± 4.5	-286.2 ± 7.9	-278.6 ± 7.5	-289.1 ± 3.2
	S. trutta	1	-33.9 ± NA	-37.8 ± NA	-34.8 ± NA	-36.1 ± NA	-301.3 ± NA	NA	-258.9 ± NA	-280.7 ± NA
**Ybbs (Kläranlage Lunz)**	*C. gobio*	7	-36.1 ± 0.8	-36.3 ± 1.7	-36.3 ± 1.3	-36.2 ± 0.8	-291.5 ± 8.5	-258.8 ± 3.9	-266.6 ± 16.1	-269.9 ± 9.1
	O. mykiss	2	-36.1 ± NA	-36.5 ± 0.4	-37.3 ± 1.4	-36.7 ± 1.2	-301.1 ± NA	-292.9 ± 1.2	-281.5 ± 29.0	-288.0 ± 1.8
	S. trutta	2	-35.5 ± 0.0	-35.2 ± 0.0	-37.2 ± NA	-36.1 ± 0.0	-304.1 ± 0.0	-279.2 ± 4.0	-281.9 ± NA	-274.1 ± 3.1
**Ybbs (Lunz Großau)**	*C. gobio*	4	-35.1 ± 0.6	-34.9 ± 0.9	-34.9 ± 0.7	-35.4 ± 0.6	-284.1 ± 2.6	-252.7 ± 4.5	-258.8 ± 17.7	-270.0 ± 7.0
	O. mykiss	7	-35.0 ± 1.0	-36.4 ± 1.7	-36.4 ± 0.5	-36.6 ± 0.9	-303.3 ± 7.2	-277.0 ± 18.7	-285.5 ± 7.0	-290.7 ± 6.7

Figure S2. Changes in fish fatty acid isotope values for (A) $\delta^{13}C$ and (B) $\delta^{2}H$ at 9 different sampling sites between 2016 and 2018.

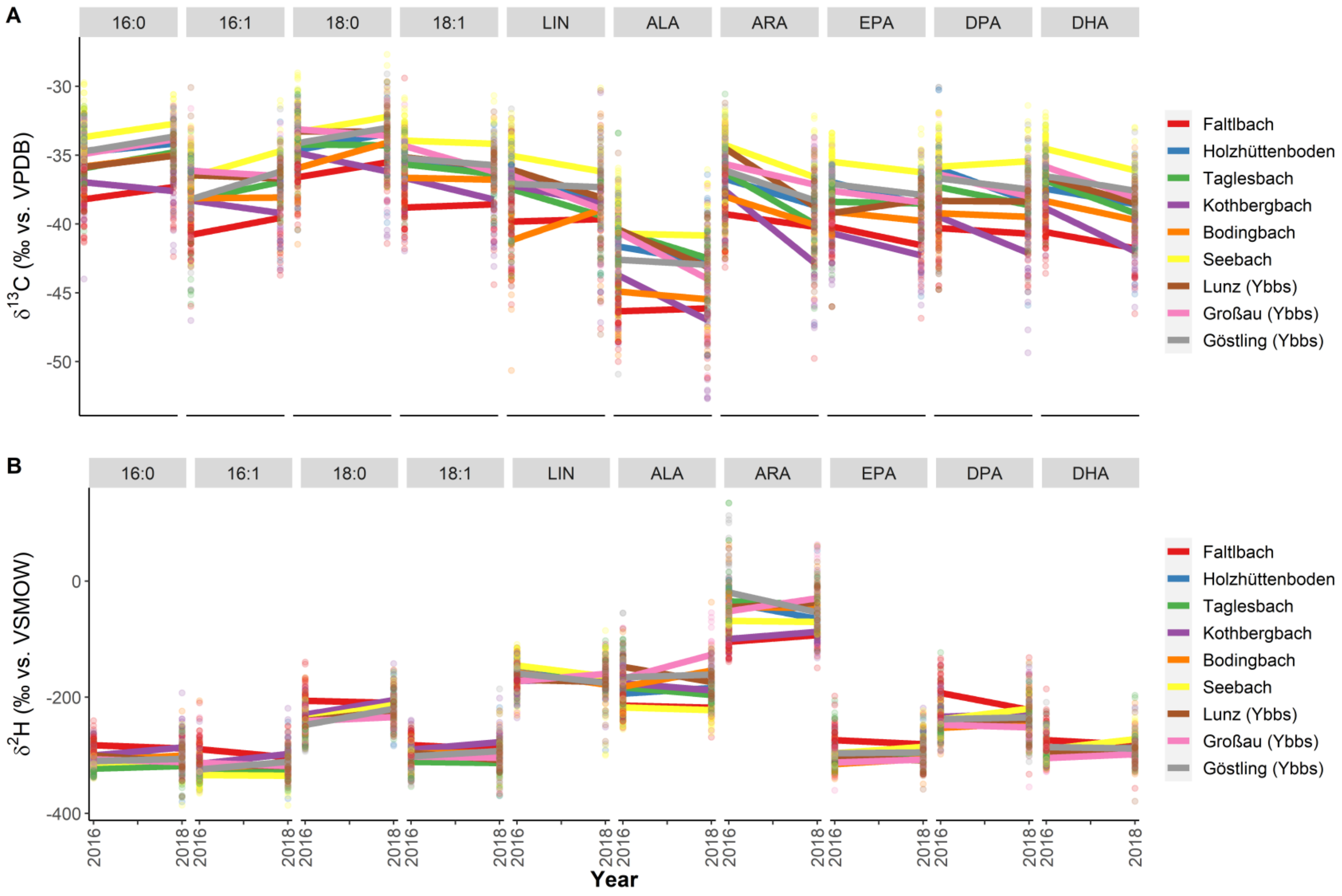

Figure S3. LDA of δ^2H and δ^{13}C values of 11 FA quantified in all samples; proportions of trace: LD1 = 48.2 %, LD2 = 23.6 %, LD3 = 7.8 %). Interactive 3Dplot of the first three dimensions of the LDA site-specificity plot downloadable as .html-file.
